# Personal Innovativeness and Digital Entrepreneurial Intention: A Social Cognitive Perspective and the Moderating Role of AI Adoption

**DOI:** 10.1186/s40359-026-05177-y

**Published:** 2026-07-22

**Authors:** Bao Quoc Lam, Hao Yen Tran, Nguyen Thi Hoa Hong

**Affiliations:** 1 Applied Economics and Business Administration Research Group (AEB), Ho Chi Minh City Open University, Ho Chi Minh City, Vietnam; 2 Faculty of Business Administration, Foreign Trade University, Ha Noi, Vietnam

**Keywords:** Digital entrepreneurial intention, Personal innovativeness, Digital entrepreneurial self-efficacy, AI adoption, Social Cognitive Career Theory

## Abstract

**Background:**

Despite the rapid growth of digital entrepreneurship and increasing adoption of artificial intelligence (AI), existing explanations of digital entrepreneurial intention (DEI) remain fragmented and inconsistent. Prior studies have primarily focused on either individual cognitive factors or technology adoption, while limited attention has been given to how AI-supported environments interact with psychological mechanisms underlying entrepreneurial intention formation. This limitation is particularly relevant in emerging economies, where digital entrepreneurship increasingly develops within technology-intensive environments.

**Methods:**

Drawing on Social Cognitive Career Theory (SCCT), this study examines the roles of personal innovativeness and digital entrepreneurial self-efficacy in shaping DEI, while also investigating the moderating role of AI adoption. Cross-sectional survey data were collected from 472 university students in Vietnam and analyzed using partial least squares structural equation modeling.

**Results:**

The findings indicate that personal innovativeness positively influences both digital entrepreneurial self-efficacy and digital entrepreneurial intention. Digital entrepreneurial self-efficacy also significantly predicts DEI and serves as an important cognitive mechanism linking innovativeness to entrepreneurial intention. Furthermore, AI adoption strengthens the relationships between innovativeness and intention, as well as between self-efficacy and intention, suggesting that AI-supported environments may enhance the translation of cognitive resources into entrepreneurial motivation.

**Conclusion:**

This study contributes to the literature by extending SCCT into digitally intensive entrepreneurial contexts and clarifying how technology-assisted learning and information environments interact with individual cognition in shaping entrepreneurial intention. The findings also provide practical implications for entrepreneurship education and AI-supported entrepreneurial learning among university students in emerging economies.

**Supplementary Information:**

The online version contains supplementary material available at https://doi.org/10.1186/s40359-026-05177-y.

## Introduction

The entrepreneurial intention of university students has long attracted scholarly attention, as it reflects the formation of entrepreneurial mindsets within higher education and signals the potential supply of future entrepreneurs [13]. In recent years, the rapid expansion of digital technologies has fundamentally reshaped entrepreneurial activities, giving rise to digital entrepreneurship as a distinct and increasingly influential phenomenon. In Vietnam, the digital economy’s revenue in 2025 was estimated at approximately USD 198 billion, representing a 26% increase compared to the previous year. Additionally, the country recorded around 80,000 digital technology enterprises in 2025, reflecting an annual growth rate of approximately 10%, [74].

Consequently, digital entrepreneurial intention (DEI), defined as individuals’ intention to initiate entrepreneurial activities primarily through digital platforms, has emerged as a critical construct in contemporary entrepreneurship research [24]. Studying DEI is important not only because it helps predict future entrepreneurial behavior, but also because it offers insights into how individuals respond to technological change and participate in the evolving digital economy [55]. This issue is particularly salient in emerging economies, where digital technologies may compensate for resource constraints and institutional voids by lowering entry barriers for new ventures. Despite its growing relevance, DEI remains a relatively underdeveloped concept, and the theoretical foundations explaining how digital entrepreneurial intentions are formed are still fragmented. Prior entrepreneurship research has tended to underemphasize both the role of digital technologies and the characteristics of individual actors engaged in digital entrepreneurship [66], resulting in a limited understanding of the mechanisms shaping DEI [48]. For instance, in the study by Li et al. [42], DEI was defined primarily in terms of the technological nature of the business, referring to individuals’ intention to adopt technology or establish technology-based ventures. However, Vu et al. [75] provided a more comprehensive perspective by incorporating not only the technological characteristics of entrepreneurship but also the internal psychological factors involved in the formation of entrepreneurial behavior. As a result, existing explanations of DEI formation remain incomplete and occasionally inconsistent, constraining theory development in this area.

Therefore, this study aims to understand the formation process of DEI further. Among behavioral theories, the social cognitive career theory (SCCT) of Lent et al. [40] is considered to have the ability to comprehensively explain the psychological processes related to behavior, especially when approaching entrepreneurship from the perspective of career choice [26]. SCCT emphasizes the critical role of self-efficacy and outcome expectation in creating the link between psychology and behavior [5]. From an entrepreneurial perspective, this theory argues that learning experiences are the decisive factor in changing the above two core factors [40]. However, SCCT does not mention the individual's learning motivation, and this makes SCCT assume that the individual passively records knowledge [39]. Meanwhile, when entrepreneurship is a complex and competence-focused behavior, proactive learning is a prerequisite and fundamental for increasing the likelihood of success when establishing a business [13].

The tendency to proactively learn is primarily determined by personality, in which innovativeness is the most prominent. In technology-based entrepreneurial contexts, innovativeness reflects an individual’s openness to new ideas, willingness to experiment with emerging technologies, and readiness to embrace change [57]. Prior research suggests that individuals with higher levels of innovativeness are more inclined to engage in exploratory learning, accumulate richer learning experiences, and develop stronger perceptions of their own abilities [34]. Accordingly, innovativeness may function as an important antecedent of entrepreneurial self-efficacy and, indirectly, digital entrepreneurial intention. While existing studies have often examined innovativeness and self-efficacy in isolation [16] or treated self-efficacy as a precursor to innovativeness, fewer studies have explored how innovativeness contributes to the development of self-efficacy within an SCCT framework. By positioning innovativeness as a driver of self-efficacy and DEI, this study seeks to extend SCCT by clarifying the motivational origins underlying proactive learning and competence development in digital entrepreneurship.

In parallel, advances in artificial intelligence (AI) have introduced a new technological context that may further shape the formation of DEI. AI technologies play an increasingly important role in improving business process efficiency, supporting decision-making, and facilitating innovation [2]. For instance, AI-powered tools such as chatbots, recommendation systems, and generative AI platforms are increasingly adopted by startups to automate customer service, personalize marketing strategies, and reduce operational costs [2]. In addition, AI applications in e-commerce and digital content creation have enabled young entrepreneurs to access broader markets, enhance productivity, and develop innovative business models with relatively limited financial resources [2].

AI's application helps students who lack entrepreneurial experience and have limited resources to increase the efficiency of their newly established ventures. Accordingly, AI adoption strengthens both confidence and the tendency to start a business [25]. Accepting and using AI accelerates the learning process and enhances its effectiveness, while also boosting confidence in entrepreneurship when AI is recognized as a valuable tool and solution for various business models [2]. The entrepreneurial intent development requires knowledge acquisition, problem-solving, and innovation, which students can pursue and develop with AI tools [82]. AI allows students to fill experience gaps and refine decision-making and idea exploration in the entrepreneurial process [82].

Despite growing scholarly interest in AI and digital entrepreneurship, empirical evidence on how AI adoption interacts with individual-level cognitive factors to shape digital entrepreneurial intention remains limited. In particular, existing studies have largely focused on the direct effects of AI adoption or digital technologies on entrepreneurial outcomes, while paying less attention to their contingent role in strengthening or weakening the influence of personal characteristics and self-beliefs. From a social cognitive career perspective, this gap is especially important, as digital entrepreneurship in emerging economies often unfolds under conditions of uncertainty, limited resources, and uneven access to entrepreneurial support. Moreover, this study extends SCCT by proposing that entrepreneurial intention is not shaped solely by internal cognitive evaluation, but also by individuals’ interaction with technology-enabled environments [23]. In this regard, AI adoption is conceptualized as a contextual catalyst that strengthens the translation of innovativeness and entrepreneurial self-efficacy into digital entrepreneurial intention [23]. By integrating personal cognition with digitally mediated learning and opportunity structures, this study offers a more nuanced understanding of entrepreneurial intention formation in technology-intensive environments [18, 20].

In response to these research gaps, this study addresses the following research questions:

- RQ1: To what extent does personal innovativeness contribute to entrepreneurial self-efficacy?
- RQ2: How does entrepreneurial self-efficacy influence digital entrepreneurial intention?
- RQ3: Does AI adoption strengthen the effects of personal innovativeness and entrepreneurial self-efficacy on digital entrepreneurial intention?

Accordingly, this study aims to examine the formation of digital entrepreneurial intention among university students by integrating personal innovativeness and entrepreneurial self-efficacy within an SCCT framework, while explicitly accounting for the moderating role of AI adoption. By conceptualizing AI adoption as a cognitive and learning catalyst, rather than a direct determinant of intention, this study seeks to clarify how digital technologies amplify the translation of innovativeness and self-efficacy into entrepreneurial intention. Furthermore, the study advances the theoretical understanding of entrepreneurship in technology-intensive settings by highlighting that entrepreneurial intention formation is shaped not only by individual traits, but also by individuals’ interaction with digitally mediated learning and opportunity structures. In doing so, the study contributes to the entrepreneurship literature by offering a more nuanced understanding of how individual cognition and emerging technologies jointly shape entrepreneurial career intentions in an emerging economy context. Beyond its relevance to entrepreneurship research, this study contributes to the psychological understanding of career-related intention formation in technology-intensive environments. By examining how personality traits and self-beliefs interact under conditions of digital tool adoption, the study provides insight into cognitive and motivational processes shaping contemporary career choices. This psychological framing is particularly important in emerging economies, where technological access may alter perceived capability and opportunity structures. Specifically, the study situates digital entrepreneurial intention within broader psychological theories of motivation and self-regulation.

The remainder of this paper is structured as follows. Sect. "Literature review and hypotheses development" reviews the relevant literature and develops the research hypotheses. Sect. "Methodology" outlines the research methodology, including data collection, measurement, and analytical procedures. Sect. "Results and discussion" presents the empirical results, followed by a discussion of the findings. Finally, Sect. "Conclusion and Implications" concludes the paper by highlighting theoretical and practical implications, acknowledging limitations, and suggesting directions for future research.

## Literature review and hypotheses development

### Digital entrepreneurial intention

Digital entrepreneurship (DE) is the initiation of a business using digital platforms enabled by the internet system through which business functions are performed entirely on digital platforms [76]. DE focuses on leveraging digital technologies, tools, or business models to explore and exploit business activities and opportunities [71]. Furthermore, DE also includes the purposeful efforts of individuals to convert business ideas into tangible products or services, which requires the development of essential processes in which digital technology plays a key role as an important input [38]. It focuses on the partial or complete digitalization of a company's products, intending to provide innovative and differentiated value propositions [14, 70]. According to Kollmann [37], DE refers to the establishment of a business with an innovative business idea in the net economy, thereby providing its products based on the creation of value entirely by technology.

Building on this foundation, digital entrepreneurial intention (DEI) captures individuals’ deliberate and conscious intention to establish a business venture whose key activities are conducted through digital platforms [8]. DEI reflects a cognitive orientation toward pursuing entrepreneurship as a digital career path, involving the willingness to initiate, organize, and manage business activities in digitally mediated environments. As a subcategory of entrepreneurial intention, DEI is distinguished by its emphasis on digital assets, digital services, and digitally enabled business models, which require specific competencies related to technology use, innovation, and adaptability [64]. Consequently, understanding DEI necessitates attention to both entrepreneurial cognition and the technological context in which entrepreneurial intentions are formed.

### Social cognitive career theory

Social Cognitive Career Theory (SCCT) provides a well-established framework for explaining how individuals develop career-related interests, intentions, and choices through the interaction of personal, behavioral, and contextual factors [40]. Rooted in Bandura’s social cognitive theorynull [11] and self-efficacy theory, SCCT conceptualizes career development as a dynamic process shaped by individuals’ beliefs about their capabilities, anticipated outcomes, and goal-setting behaviors. More recently, SCCT has been increasingly applied to entrepreneurship research, as entrepreneurship is commonly viewed as a distinct career choice rather than a purely economic activity [26]. Recent studies further support the relevance of SCCT in explaining entrepreneurial intention formation. Soomro and Shah [63] found that self-efficacy and outcome expectations significantly influence entrepreneurial intention among university students, while Duong [23] demonstrated that SCCT effectively explains technology-oriented entrepreneurial intention through the interaction between cognitive and environmental mechanisms. These findings suggest that SCCT is particularly suitable for understanding entrepreneurial intention in digitally intensive and technology-driven environments.

Compared with other intention-based frameworks, particularly the Theory of Planned Behavior (TPB), SCCT provides a more comprehensive explanation of entrepreneurial intention formation in technology-intensive contexts. While TPB primarily explains intention through attitudes, subjective norms, and perceived behavioral control, SCCT places stronger emphasis on self-efficacy, learning experiences, and the interaction between individual cognition and contextual conditions [40]. This perspective is particularly relevant for digital entrepreneurship, where entrepreneurial decisions are strongly influenced by technology-assisted learning environments, access to information, and rapidly evolving digital opportunities [19].

With regard to SCCT, there are three key framework constructs including self-efficacy, anticipated results, and objectives. These constructs clarify how individuals navigate their career choices [15]. SCCT incorporates aspects from vocational psychology to offer a more organized explanation of one’s personal experiences and perceptions towards a career, and the ‘decisions’ involved. This also highlights and enhances the importance of the individual’s personal agency and human potential [5]. It clarifies the consolidation of interest and the decision-making actions undertaken in a particular career [35].

In addition, SCCT incorporates the interaction of personal, situational, and behavioral dimensions in explaining entrepreneurial career interests [41]. Prior studies suggest that self-efficacy positively influences entrepreneurial intention because individuals who perceive themselves as capable are more likely to pursue entrepreneurial opportunities and persist in uncertain environments [17, 28]. At the same time, recent studies increasingly argue that entrepreneurial intention is not solely determined by internal cognition, but also shaped by broader contextual conditions such as entrepreneurial ecosystems, institutional support, digital infrastructure, and technology-enabled learning environments [23, 69, 71]. This perspective is particularly important in emerging economies, where access to digital technologies and entrepreneurial resources may significantly influence how entrepreneurial cognition is translated into intention [19].

Despite the growing use of SCCT in entrepreneurship research, prior studies often examine psychological determinants, personality traits, and technological conditions separately, resulting in fragmented explanations of digital entrepreneurial intention [23, 69, 71]. Furthermore, limited attention has been given to how innovativeness and AI-supported environments interact to strengthen entrepreneurial self-efficacy and entrepreneurial intention simultaneously. Therefore, this study extends SCCT by integrating personal innovativeness, digital entrepreneurial self-efficacy, and AI adoption into a unified framework to explain digital entrepreneurial intention in digitally intensive environments.

### Digital entrepreneurial self-efficacy

Self-efficacy is the capacity of individuals to assess their ability to carry out relevant actions to fulfill the requirements of a particular setting [45]and the ability of individuals to complete and execute the organization and planning of a set of actions [12]. It also involves personal beliefs concerning the ability to execute certain actions, which are different and tailored to particular activities [58]. On the other hand, entrepreneurial self-efficacy is the belief of a person concerning their ability to perform entrepreneurial activities pertaining to starting and running a particular project [53]. Such beliefs are drawn from the SCCT, which maintains that individuals develop an interest in and are likely to excel in activities where they have strong self-efficacy beliefs, requisite skills, and environmental encouragement to partake in these activities [58].

Within the DE context, digital entrepreneurial self-efficacy means the conviction to operate as a digital entrepreneur competently [79]. SCCT posits that individuals with self-efficacy are more likely to participate in activities they believe they can accomplish (successful). The complex digital business environment demonstrates the extent to which digital entrepreneurial self-efficacy is mastered. Furthermore, the perceived silos of digital business pivots, technology, and market mastery fuel entrepreneurial self-efficacy [14]. Digital entrepreneurial business opportunities are pursued by self-efficacy holders as they easily overcome obstacles, self-initiate, and take entrepreneurship. Risk management and problem-solving skills drive project completion. Such individuals are most likely to engage with entrepreneurial ideas.

Existing studies consistently identify entrepreneurial self-efficacy as one of the strongest predictors of entrepreneurial intention [33, 68]. However, much of the existing literature primarily treats self-efficacy as an isolated cognitive predictor, while paying less attention to how self-efficacy is strengthened through technology-enabled learning experiences and innovative behavioral tendencies [55, 61]. Recent studies suggest that digital environments, AI-supported learning systems, and technology-assisted information access may significantly enhance individuals’ perceived entrepreneurial capability by reducing uncertainty and facilitating knowledge acquisition [14, 71].

Moreover, in digital entrepreneurship settings, individuals with stronger digital entrepreneurial self-efficacy are more likely to recognize digital opportunities, overcome technological barriers, and persist in uncertain entrepreneurial environments [19, 55]. This relationship may be especially important in emerging economies, where entrepreneurial ecosystems and institutional support structures are still evolving, thereby increasing the importance of individual cognitive confidence in entrepreneurial decision-making [10]. Therefore, digital entrepreneurial self-efficacy is expected to positively influence digital entrepreneurial intention.

- H1: Digital entrepreneurial self-efficacy positively impacts digital entrepreneurial intention

### Personal innovativeness

Personal innovativeness has been widely examined as a core individual characteristic in entrepreneurship research [4]. It is commonly defined as an individual’s tendency to adopt new ideas, technologies, or innovations earlier than others within a social system [60]. It is a critical feature of entrepreneurship because it includes opportunity recognition, idea development, and innovation [80], the ability to create Valuable Novelties, which involves testing, observing, and analyzing, and the ability to predict [65]. Also, personal innovativeness is a characteristic that augments the understanding of the entrepreneurial [67]. Thus, the innovativeness of entrepreneurial activities sustains business operations by creating, adjusting, and applying the concept of value within ideas.

Entrepreneurs utilize their originality to address issues, create new concepts, and pinpoint answers. Personal innovativeness, which is linked to entrepreneurship, is pivotal for entrepreneurial achievements [46]. This capacity to find answers, address issues, and develop new offerings contributes to entrepreneurial aspirations and growth, which entails productivity, efficiency, and competitive edge. There are core similarities between traditional and digital entrepreneurship [48], but the former is more dynamic and adaptable because of rapid changes in digital technology and the attributes of the digital products or services offered [4, 49]. Hence, personal innovativeness will significantly assist in delineating and recognizing the prospects in digital entrepreneurship and the intention to pursue digital entrepreneurship.

Entrepreneurship is possible for anyone with some imagination, enthusiasm, and a willingness to take risks [77]. Moreover, previous research has identified innovativeness as a key determinant with a positive correlation to entrepreneurial intention [7, 52, 54, 81]. It forms a vital part of one’s personality, facilitating digital entrepreneurship [47] and exerting a considerable impact on entrepreneurial intention [59]. Here, the hypothesis is argued as follows:

- H2: Personal innovativeness has a positive effect on digital entrepreneurial intention

Beyond its direct effect on intention, personal innovativeness may also shape entrepreneurial self-efficacy. Innovativeness primarily involves recognizing and implementing ideas rather than merely generating them [22]. Nambisan et al. [50] states that in the digital era, personal innovativeness encompasses the pursuit of innovations extending beyond the boundaries of traditional industries. Currently, personal innovativeness in the digital era encompasses innovations through the use of digital technologies and the pursuit of ideas in the integrated advanced technologies. This is often perceived as recognition of an opportunity coupled with exclusiveness, thus constituting an intellectual monopoly with augmentation of personal confidence.

In the absence of creativity, digital entrepreneurial self-efficacy is said to reside in personal innovativeness [3]. Increased creativity and confidence lead to adopting new technologies and experiences. Furthermore, personal innovativeness enhances the perception of self-capability and self-creation which increases self-efficacy [51]. Therefore, creativity enables learners to facilitate their learning outcomes, skills, and knowledge by adopting new learning technologies and systems [3].

- H3: Personal innovativeness positively influences digital entrepreneurial self-efficacy

### AI adoption

Among emerging and disruptive technologies, artificial intelligence (AI) has the potential to be a game changer [73]. It not only accelerates technological and economic growth, but helps businesses formulate and implement more effective strategies, improve the delivery of services, and reduce costs [78]. However, within the context of student entrepreneurship, AI primarily functions as a learning support and cognitive enhancement tool [82]. Previous studies suggest that AI technologies can help individuals access relevant information more efficiently, enhance knowledge development, and support opportunity exploration in entrepreneurial environments [82],[24, 25]. For university students and young entrepreneurs, AI-supported tools such as generative AI systems, intelligent search assistants, and personalized learning platforms can facilitate idea generation, entrepreneurial learning, and problem-solving activities [24]. In addition, AI can monitor and influence business models, facilitate the innovation of business processes, and assist in the consolidation of operational processes [2]. The integration of AI in business also helps in the adoption of advanced paradigms, allowing business owners to leverage technology for rapid and significant growth [43]. Above all, AI’s fundamental importance lies in helping business owners develop new ways and new opportunities to solve difficult business problems [29].

Within the field of digital entrepreneurship, the role of AI continues to expand, notably in the formulation of innovative business models that leverage digital technologies [27]. It helps entrepreneurs forecast certain business trends and aids rational decision-making during the exploration and innovative phases of evaluating business ideas [69]. Innovation has been described as an accelerator, and with the rapid pace of AI, the ability of entrepreneurs to access key resources and information to perform their entrepreneurial activities has been significantly enhanced [72]. Young entrepreneurs derive higher efficiency from personal innovativeness in the startup ecosystem due to the integration of AI into their business activities [6]. Most importantly, AI reduces barriers to information access and enhances individuals’ ability to acquire knowledge [82]. In organizational and educational contexts, learning-supportive environments have been shown to positively influence individuals' cognitive development and performance outcomes [56], suggesting that AI-enabled learning tools may similarly strengthen entrepreneurial cognition among university students. AI may also strengthen the positive effects of personal innovativeness on entrepreneurial cognition and exploratory behaviors. Individuals with high levels of personal innovativeness are generally more willing to experiment with emerging technologies and actively seek new knowledge [57]. When supported by AI-based information and learning tools, these individuals may further increase their confidence in exploring digital business opportunities and transforming innovative ideas into entrepreneurial intentions [21]. The synergistic effect of advanced technologies, innovative business ideas, and AI capabilities, substantially increases the chances of an individual to form entrepreneurial intentions.

- H4: AI adoption moderates the relationship between personal innovativeness and digital entrepreneurial intention

In addition, SCCT suggests that AI use may enhance perceived efficacy beliefs among individuals considering digital entrepreneurship, which affects their digital entrepreneurial intentions [14]. AI-supported systems can provide personalized feedback, rapid access to entrepreneurial knowledge, and learning assistance, which may enhance individuals’ perceptions of their entrepreneurial capabilities. Prior studies have shown that digital learning support and technology-enabled knowledge acquisition positively influence entrepreneurial self-efficacy [1, 71]. In this regard, AI adoption may not directly determine entrepreneurial intention, but instead function as a cognitive support mechanism that strengthens the effect of entrepreneurial self-efficacy on digital entrepreneurial intention [30]. Furthermore, AI-assisted learning and information accessibility may reduce uncertainty and increase confidence in performing entrepreneurship-related tasks in digital environments.

- H5: AI adoption moderates the relationship between digital entrepreneurial self-efficacy and digital entrepreneurial intention.

**Fig. 1:**
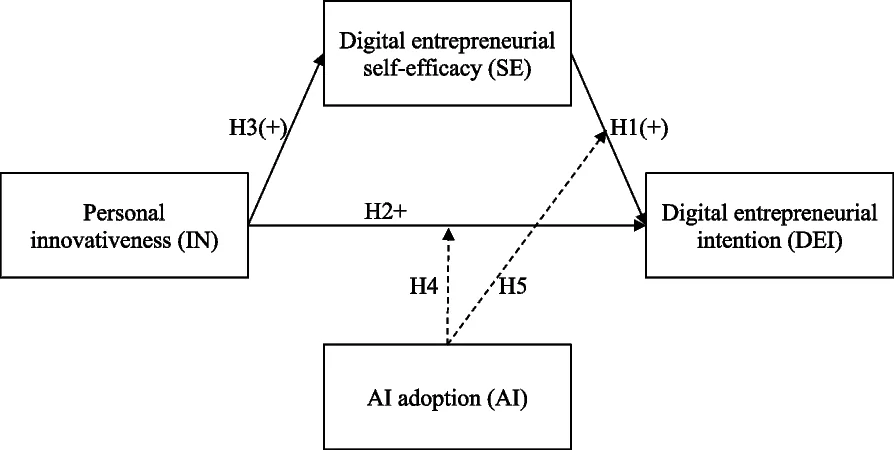
Research model

Figure 1 presents the proposed research model and hypotheses*.*

## Methodology

This study was conducted in Vietnam and focused on university students enrolled in multiple higher education institutions. University students represent an appropriate population for examining digital entrepreneurial intention, as they are at an early stage of career decision-making and are actively forming entrepreneurial aspirations [55]. Moreover, students typically possess foundational knowledge in management, entrepreneurship, or technology-related fields and are more exposed to digital tools and emerging technologies such as artificial intelligence. This makes them particularly relevant for investigating how cognitive factors and AI adoption jointly shape digital entrepreneurial intention in an emerging economy context.

### Data collection and sample

Vietnam was selected as the research context due to its dynamic entrepreneurial environment, rapid digital transformation, and increasing integration of AI technologies into education and business activities [74]. In recent years, Vietnam has witnessed a significant rise in startup support programs, digital innovation initiatives, and AI-related applications among university students and young entrepreneurs. Moreover, the growing accessibility of digital platforms and AI-powered tools has created favorable conditions for young people to explore entrepreneurial opportunities in the digital economy [24, 25]. Therefore, Vietnam provides an appropriate and relevant context for examining the factors influencing DEI among university students.

Data were collected between October and November 2025 using a convenience sampling approach through an online questionnaire administered via Google Forms. The use of convenience sampling was considered appropriate for this study because the target population consisted of university students from different academic disciplines and institutions, making probability sampling difficult due to the lack of a comprehensive sampling frame. In addition, online survey distribution enabled broader access to respondents who are highly familiar with digital technologies and AI-related applications, which aligns with the focus of this study on digital entrepreneurship and AI adoption. Convenience sampling has also been widely employed in prior entrepreneurship, technology acceptance, and higher education studies due to its practicality, cost efficiency, and suitability for exploratory behavioral research.

The questionnaire consisted of three sections. The first section provided an overview of the study objectives, assured respondents of confidentiality and anonymity, and included screening questions to ensure participant eligibility. The second section contained measurement items for the study’s main constructs, which were adapted from previously validated scales and culturally adjusted to the Vietnamese context. The final section captured demographic information, including gender, academic discipline, and year of study.

A total of 500 responses were initially collected. After excluding incomplete or invalid entries, 472 valid responses were retained for analysis. Among the respondents, 302 were female (63.9%) and 170 were male (36.1%). With respect to academic discipline, 224 participants (47.5%) were enrolled in Economics and Business programs, 130 (27.5%) in Engineering, and 118 (25.0%) in other fields such as Biotechnology, Medicine, and Information Technology. Regarding academic standing, 95 respondents (20.1%) were first-year students, 186 (39.4%) were second-year students, 142 (30.1%) were third-year students, and 49 (10.4%) were final-year students.

### Items measurement

All constructs in the study were measured using multi-item scales adapted from prior research to ensure content validity. Specifically, Digital Entrepreneurial Intention (DEI), Digital Entrepreneurial Self-efficacy (SE), and Personal Innovativeness (IN) were measured using previously validated scales developed by Pham et al. [55], while AI adoption (AI) was measured using five items adapted from Duong [24]. The DEI scale consisted of five items (e.g., “My professional goal is to become an entrepreneur on digital platforms”), the SE scale included six items (e.g., “I am confident in digital-entrepreneurship”), the IN scale comprised three items (e.g., “I like to experiment with new information technology”), and the AI adoption scale contained five items assessing the use of AI tools to obtain entrepreneurial information and support decision-making.

All measurement items used in this study were previously published in peer-reviewed journals. Minor wording adjustments were made solely to ensure contextual appropriateness for Vietnamese university students, without altering the conceptual meaning of the original items. The full English version of the questionnaire, including all measurement items and construct operationalization, is provided as Supplementary File 1.

Responses were recorded on a five-point Likert scale ranging from 1 being “Strongly disagree” to 5 being “Strongly agree”. The use of a consistent Likert scale across constructs facilitated comparability and minimized respondent confusion. The measurement items were pre-tested to ensure clarity and contextual appropriateness before full-scale data collection.

### Ethical considerations

The study was conducted with ethical approval from Ho Chi Minh City Open University, granted on July 24, 2025, and followed the ethical principles outlined in the Declaration of Helsinki. Participation in the survey was entirely voluntary, and respondents were informed of their right to withdraw at any time without penalty. All data were collected anonymously and used solely for academic research purposes. Confidentiality of respondents’ information was strictly maintained throughout the research process.

### Data analysis

Data analysis was conducted following the two-step procedure recommended by Hair et al. [31] for partial least squares structural equation modeling (PLS-SEM). PLS-SEM was deemed appropriate for this study given its suitability for theory extension, prediction-oriented research, and models incorporating interaction effects. Notably, CB-SEM is not suitable for models with product indicator-based interaction terms, as it produces inadmissible structural results in such contexts [31]. To further verify the measurement model, a CB-SEM algorithm was additionally applied for comparison, with results reported in Supplementary File 2. In the first step, the measurement model was assessed to evaluate internal consistency and convergent validity using Cronbach’s Alpha (CA), rho_A, Composite Reliability (CR), Average Variance Extracted (AVE), and outer loadings. Discriminant validity was examined using the Heterotrait-Monotrait (HTMT) ratio. In the second step, the structural model was evaluated by examining multicollinearity through variance inflation factors (VIF), explanatory power using R^2^ values, and predictive relevance using Q^2^ statistics. To test the significance of the hypothesized relationships, a bootstrapping procedure with 5,000 resamples was employed to generate path coefficients, t-values, and confidence intervals.

To assess potential common method bias, Harman’s single-factor test was conducted. The first unrotated factor accounted for less than 50% of the total variance, indicating that common method bias was unlikely to pose a serious concern. In addition, full collinearity variance inflation factors (VIFs) were examined and remained below recommended thresholds, further reducing concerns regarding method bias.

## Results and discussion

### Measurement model

The measurement model was assessed to examine internal consistency reliability, convergent validity, and discriminant validity. Reliability was evaluated using Cronbach’s alpha (CA), rho_A, and composite reliability (CR), while convergent validity was assessed through average variance extracted (AVE) and indicator outer loadings. As shown in Table 1, all constructs meet the recommended thresholds suggested by Hair et al. [31]. Specifically, all CA and CR values exceed the minimum criterion of 0.70, indicating satisfactory internal consistency. The lowest CA value was observed for Innovativeness (0.769), and the lowest CR value was recorded for AI adoption (0.868), both well above acceptable levels. Additionally, all rho_A values range from 0.770 to 0.847, falling between Cronbach's alpha and composite reliability as expected, and confirming the reliability of all constructs [31]. All AVE values are greater than 0.50, ranging from 0.565 for Self-efficacy to 0.685 for Innovativeness, confirming adequate convergent validity. In addition, all indicator outer loadings exceed the recommended threshold of 0.70, suggesting that the observed variables reliably represent their respective latent constructs.

**Table 1:** Measurement criteria

Variables	CA	rho_A	CR	AVE	Outer loading
AI adoption (AI)	0.810	0.813	0.868	0.568	0.737—0.769
Digital entrepreneurial intention (DEI)	0.825	0.834	0.876	0.587	0.743—0.789
Personal innovativeness (IN)	0.769	0.770	0.867	0.685	0.805—0.851
Digital entrepreneurial self-efficacy (SE)	0.846	0.847	0.886	0.565	0.740—0.764

Discriminant validity was examined using the heterotrait–monotrait (HTMT) ratio. As shown in Table 2, all HTMT values are below the conservative threshold of 0.85 [32], indicating that the constructs are empirically distinct. In addition, the Fornell–Larcker results further confirm the distinctiveness of the constructs, as the square root of the AVE for each construct is higher than its correlations with other constructs. Specifically, the diagonal values for AI adoption (0.754), DEI (0.766), personal innovativeness (0.827), and Self-efficacy (0.752) exceed the corresponding inter-construct correlations, demonstrating that each construct shares more variance with its own indicators than with other constructs in the model [29]. Overall, both HTMT and Fornell–Larcker criteria provide consistent evidence supporting adequate discriminant validity of the measurement model.

**Table 2:** Discriminant validity

HTMT					Fornell-Larcker			
	**AI**	**DEI**	**IN**	**SE**	**AI**	**DEI**	**IN**	**SE**
**AI**					0.754			
**DEI**	0.409				0.344	0.766		
**IN**	0.308	0.499			0.245	0.407	0.827	
**SE**	0.215	0.540	0.468		0.179	0.457	0.381	0.752

*Note: AI* - AI adoption, *DEI* - Digital entrepreneurial intention, *IN* - Personal innovativeness, *SE* - Digital entrepreneurial self-efficacy

### Structural model

Following the evaluation of the measurement model, the structural model was assessed to examine collinearity, explanatory power, and predictive relevance. Multicollinearity was evaluated using variance inflation factors (VIF). As reported in Table 3, all VIF values range from 1.464 to 1.794, well below the conservative threshold of 5, indicating no serious multicollinearity concerns among the predictor variables [31].

**Table 3:** Structural model assessment

	**VIF**	**R**^**2**^	**Q**^**2**^	**Q**^**2**^**_predict**
AI adoption (AI)	1.533—1.615			
Digital entrepreneurial intention (DEI)	1.610—1.794	0.393	0.282	0.222
Personal innovativeness (IN)	1.464—1.716			
Digital entrepreneurial self-efficacy (SE)	1.587- 1.792	0.145	0.137	0.079

Effect size (f^2^) results in Table 4 indicate that personal innovativeness has a small effect on digital entrepreneurial intention (f^2^ = 0.045) and a moderate effect on entrepreneurial self-efficacy (f^2^ = 0.170). Entrepreneurial self-efficacy also shows a small effect on intention (f^2^ = 0.093), while AI adoption has a small effect on digital entrepreneurial intention (f^2^ = 0.116). Overall, innovativeness exerts the strongest effect within the model through its moderate influence on self-efficacy, while the remaining relationships contribute at smaller but meaningful levels.

**Table 4:** Effect sizes

	AI adoption (AI)	Digital entrepreneurial intention (DEI)	Personal innovativeness (IN)	Digital entrepreneurial self-efficacy (SE)
AI adoption (AI)		0.116		
Digital entrepreneurial intention (DEI)				
Personal innovativeness (IN)		0.045		0.170
Digital entrepreneurial self-efficacy (SE)		0.093		

The coefficient of determination (R^2^) for Digital Entrepreneurial Intention (DEI) is 0.393, indicating that approximately 39.3% of the variance in DEI is explained by the model. This level of explanatory power is considered moderate and acceptable in behavioral and entrepreneurship research. Meanwhile, the R^2^ value for Self-efficacy is 0.145, suggesting a modest yet meaningful level of explanation, consistent with the role of innovativeness as a distal antecedent in SCCT-based models. Predictive relevance was assessed using the Stone–Geisser Q^2^ statistic. The Q^2^ values for DEI (0.282) and Self-efficacy (0.137) are both above zero, confirming that the model exhibits satisfactory predictive relevance [31]. To further evaluate out-of-sample predictive power, PLSpredict was conducted with an omission distance of 7. The Q^2^_predict values for Digital Entrepreneurial Intention (0.222) and Digital Entrepreneurial Self-efficacy (0.079) are both positive, indicating that the model generates meaningful predictions beyond simply using the average as a benchmark [62].

### Common method bias (CMB) test

Common method bias (CMB) was assessed using Harman’s single-factor test in SPSS 20.0. The first factor accounted for 30.200% of the total variance, which is below the recommended threshold of 50% ). This suggests that common method bias is unlikely to be a serious concern in this study. In addition, all VIF values were below 3.3, meeting the criterion suggested by Kock [36] and further indicating that CMB does not significantly affect the model.

### Results and discussion

**Fig. 2:**
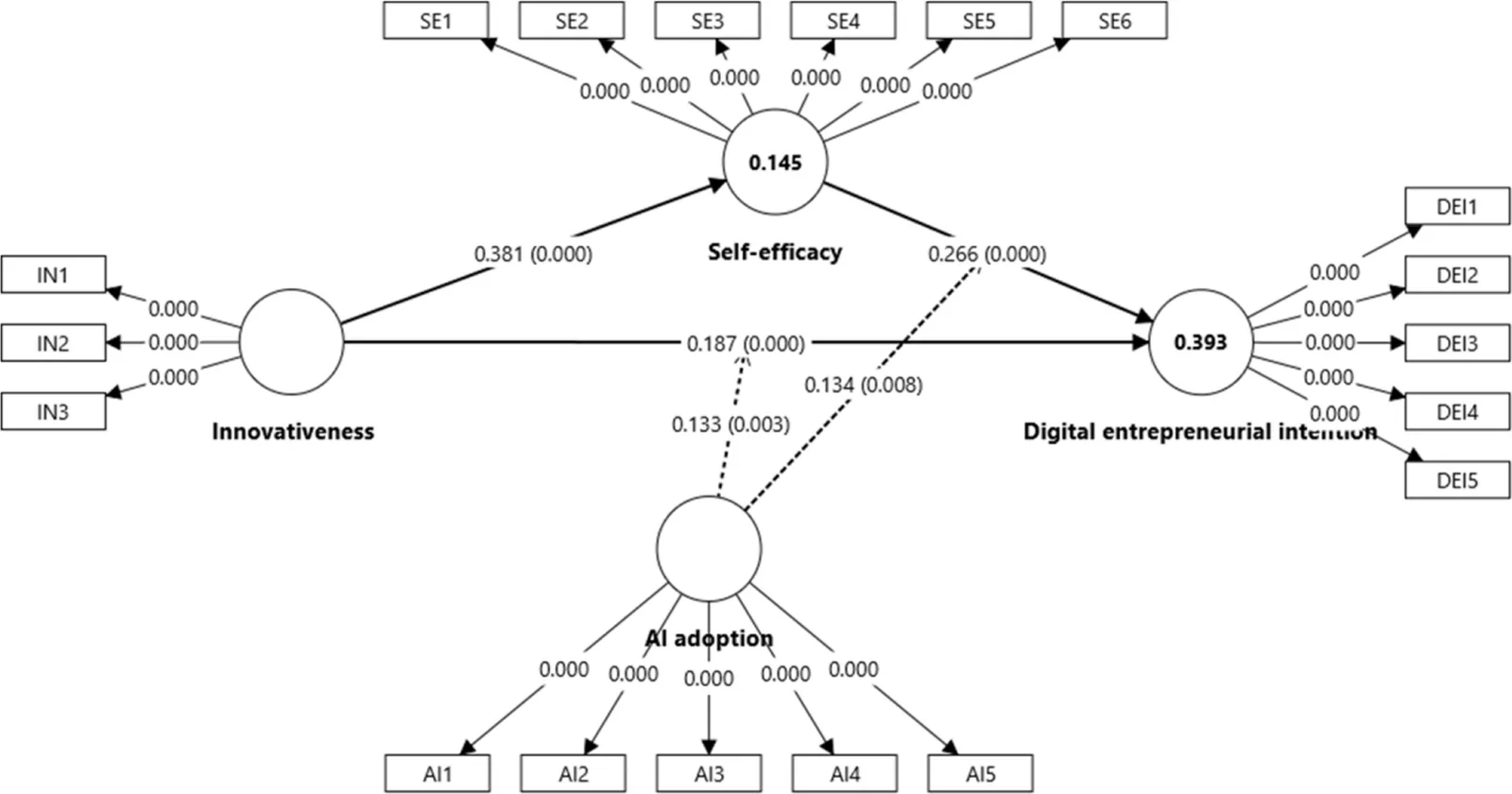
SEM result

The results of the structural model and hypothesis testing are presented in Table 5, while the estimated structural relationships are illustrated in Fig. 2. The findings indicate that all hypothesized relationships in the proposed model are supported. Digital entrepreneurial self-efficacy and personal innovativeness are both positively associated with digital entrepreneurial intention. In addition, personal innovativeness shows a positive relationship with digital entrepreneurial self-efficacy. The results further reveal that AI adoption moderates the relationships between personal innovativeness and digital entrepreneurial intention, as well as between digital entrepreneurial self-efficacy and digital entrepreneurial intention.

**Table 5:** Hypothesis testing results

Relationship	Hypo	β	T statistic	P value	Result
Self-efficacy (SE)—> Digital entrepreneurial intention (DEI)	H1	0.266	4.855	0.000	Accepted
Innovativeness (IN)—> Digital entrepreneurial intention (DEI)	H2	0.187	3.780	0.000	Accepted
Innovativeness (IN)—> Self-efficacy (SE)	H3	0.381	7.274	0.000	Accepted
AI adoption x Innovativeness (AI x IN)—> Digital entrepreneurial intention (DEI)	H4	0.133	2.956	0.003	Accepted
AI adoption x Self-efficacy (AI x SE)—> Digital entrepreneurial intention (DEI)	H5	0.134	2.644	0.008	Accepted

The findings of this study provide empirical support for the proposed framework grounded in Social Cognitive Career Theory in explaining digital entrepreneurial intention among university students. From a psychological perspective, the results clarify how personality-based dispositions and efficacy beliefs interact to shape intention formation in a digitally mediated career context. Digital entrepreneurial self-efficacy emerges as a central cognitive mechanism shaping individuals’ intention to pursue digital entrepreneurship. This result reinforces prior evidence that entrepreneurial intention in digital contexts is strongly anchored in individuals’ confidence in their ability to manage entrepreneurial tasks, technological complexity, and uncertainty [52, 81]. The finding is also consistent with SCCT, which emphasizes self-efficacy as a key determinant translating personal cognition into career-related intention and behavioral motivation [40].

Personal innovativeness also plays an important role in the formation of digital entrepreneurial intention. Individuals who are more open to experimentation, novelty, and technological change are more inclined to consider digital entrepreneurship as a viable career path. At the same time, innovativeness contributes substantially to the development of digital entrepreneurial self-efficacy, indicating that innovative individuals are more likely to engage in proactive learning and experimentation that enhance their perceived entrepreneurial capabilities [51, 55]. This finding suggests that innovativeness operates as a dispositional cognitive resource that facilitates the accumulation of mastery-related experiences, thereby strengthening efficacy beliefs. This result aligns with previous entrepreneurship studies suggesting that innovative individuals are more likely to seek new knowledge, experiment with technologies, and develop stronger confidence in entrepreneurial problem-solving activities [3, 55].

A closer examination of the relationships reveals a cognitive distinction between innovativeness and self-efficacy. Innovativeness appears to contribute more strongly to the development of perceived capability than to the direct formation of digital entrepreneurial intention. In contrast, digital entrepreneurial self-efficacy functions as a more immediate cognitive foundation for entrepreneurial decision-making. This pattern is consistent with the SCCT perspective, which emphasizes self-efficacy as the primary mechanism through which personal characteristics are translated into career-related intentions [14, 79]. In other words, personality-related tendencies appear to influence intention largely through their impact on individuals’ perceived capability to perform relevant career behaviors.

From a cognitive standpoint, innovativeness may foster optimism, risk tolerance, and readiness to engage with new technologies, thereby shaping positive expectations regarding digital entrepreneurial outcomes [55]. However, such expectations may be less stable unless they are supported by a strong sense of perceived capability. Digital entrepreneurial self-efficacy reflects a more task-oriented belief system grounded in perceived mastery of entrepreneurial activities. Accordingly, innovativeness appears to foster digital entrepreneurial intention more effectively when it is embedded within self-efficacy, highlighting the complementary roles of personality-related and capability-based cognition in entrepreneurship. This complementary relationship underscores the importance of examining both distal personality traits and proximal cognitive beliefs when explaining intention formation.

**Fig. 3:**
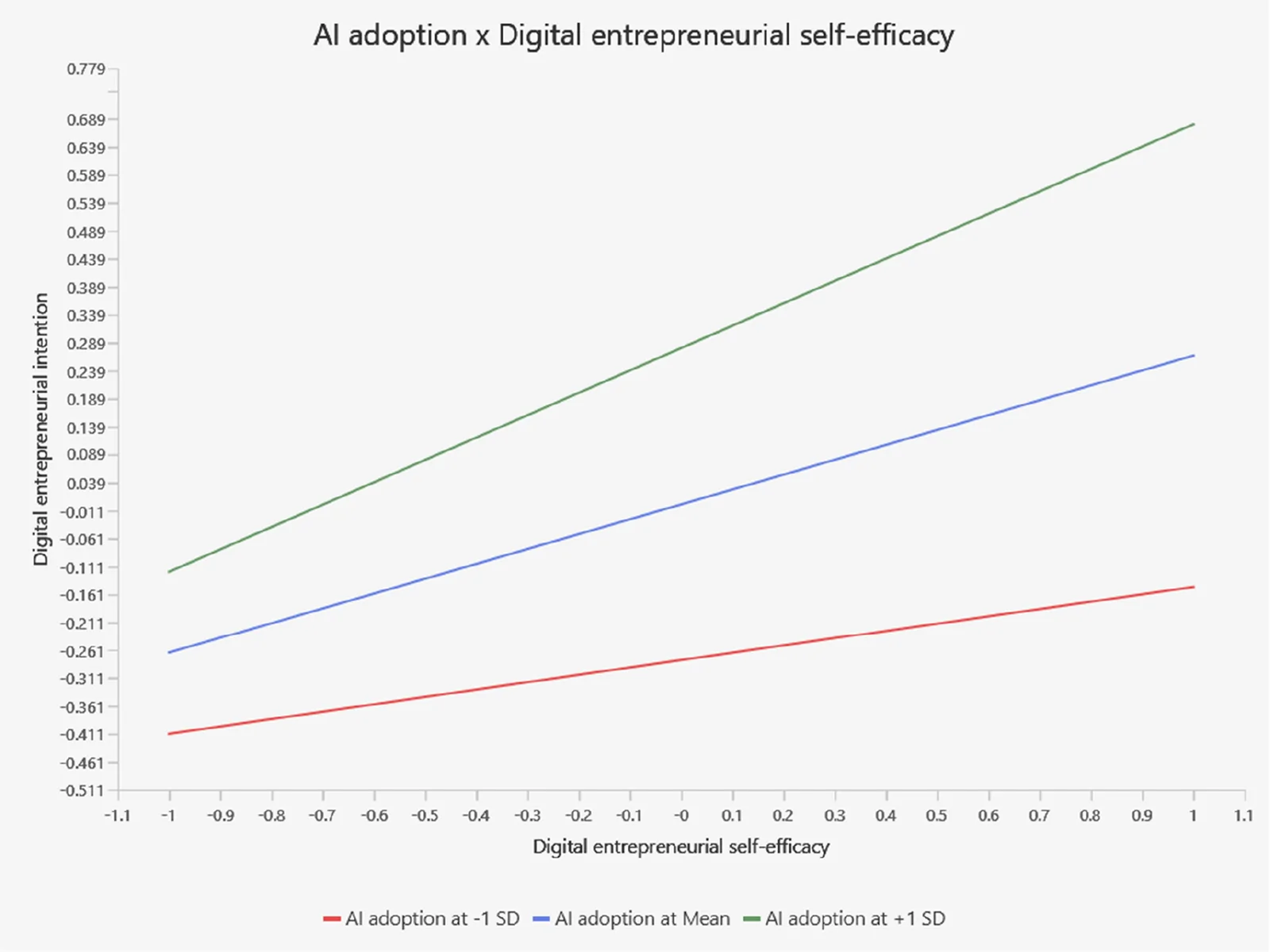
Simple slope analysis of the moderating effect of AI adoption

The moderating role of AI adoption further clarifies this cognitive process. AI adoption strengthens the relationships between both innovativeness and digital entrepreneurial intention, and between digital entrepreneurial self-efficacy and digital entrepreneurial intention. This finding suggests that AI functions as a contextual enabler that enhances the efficiency with which cognitive resources are translated into entrepreneurial intention. The simple slope analysis in Fig. 3 further demonstrates that these positive relationships become stronger under higher levels of AI adoption. Unlike earlier studies that mainly treated AI as a direct technological driver of entrepreneurial outcomes [9, 44], the present findings suggest that AI primarily functions as a cognitive and learning support mechanism within student entrepreneurship contexts.

For university students and young entrepreneurs, AI-supported tools such as generative AI systems, intelligent search assistants, and personalized learning platforms may facilitate entrepreneurial learning, improve access to relevant information, and support opportunity exploration [82]. In this sense, AI adoption helps reduce informational and cognitive barriers associated with digital entrepreneurship. Rather than acting as a direct determinant, AI adoption appears to condition the strength of belief–intention relationships, amplifying the motivational effects of innovativeness and self-efficacy. When individuals actively adopt AI tools, innovative tendencies can be more effectively transformed into entrepreneurial motivation, as AI supports experimentation, learning, and decision-making [69, 72]. This finding is consistent with prior studies arguing that AI-enabled learning environments can strengthen entrepreneurial cognition by improving knowledge development and problem-solving capability [72].

Similarly, AI adoption reinforces the role of self-efficacy by lowering perceived barriers to entrepreneurial action. Through learning facilitation, problem-solving support, and access to information, AI strengthens individuals’ confidence in managing digital entrepreneurial tasks, thereby facilitating the conversion of perceived capability into intention. This result also supports the SCCT perspective that contextual and technological conditions may strengthen the relationship between efficacy beliefs and career intention formation. In this sense, AI may operate as a contextual cognitive amplifier within technology-intensive environments.

This discussion highlights how digital entrepreneurial intention is shaped by the interaction between individual cognition and technological context. By integrating personal innovativeness, entrepreneurial self-efficacy, and AI adoption within an SCCT framework, the study clarifies how emerging technologies condition entrepreneurial career intentions in digitally intensive and emerging economy contexts. More broadly, the findings contribute to psychological research on career intention by demonstrating how technology use may modify the translation of dispositional and efficacy-based cognition into goal-directed behavioral intention.

## Conclusion and implications

### Theoretical implication

This study contributes to the literature on career-related intention formation in technology-intensive contexts by clarifying the cognitive mechanisms underlying digital entrepreneurial intention within a Social Cognitive Career Theory framework. The findings indicate that personal innovativeness plays an important role in shaping both digital entrepreneurial self-efficacy and digital entrepreneurial intention. Rather than challenging SCCT, these results extend its application by highlighting how individual-level characteristics may operate alongside learning experiences to support the development of self-efficacy. In this regard, innovativeness can be viewed as a dispositional cognitive resource that facilitates proactive engagement with learning and experimentation, thereby strengthening perceived entrepreneurial capability.

The results also reinforce the central role of digital entrepreneurial self-efficacy as a proximal determinant of digital entrepreneurial intention. Consistent with the logic of SCCT, self-efficacy serves as a key mechanism through which personal characteristics and experiences are translated into career-related intentions. By positioning innovativeness as a distal antecedent that contributes to the development of self-efficacy, this study offers a more nuanced understanding of how personality-related cognition and capability-based beliefs interact in the formation of digital entrepreneurial intention. This distinction between distal traits and proximal efficacy beliefs contributes to psychological research on how intentions emerge from layered cognitive processes.

In addition, the study contributes to the emerging literature on artificial intelligence and digital entrepreneurship by clarifying the role of AI adoption as a contextual condition in the intention formation process. The findings suggest that AI adoption does not operate as a direct driver of digital entrepreneurial intention but instead strengthens the translation of innovativeness and self-efficacy into intention. By conceptualizing AI adoption as a moderating factor, this study advances current discussions on human–technology interaction in entrepreneurship and highlights the importance of considering how technological contexts shape the effectiveness of individual cognition in career decision-making. More broadly, the results illustrate how technology use may condition the strength of belief–intention relationships, thereby enriching psychological perspectives on technology-mediated motivation.

However, this study does not seek to provide a comprehensive extension of the SCCT framework. Instead, it aims to offer a deeper understanding of how individuals interact with digitally mediated environments, particularly in the AI era, and how such interactions influence the formation of digital entrepreneurial intention (DEI). In this sense, the findings provide a foundation for future research to integrate multi-level contextual supports and entrepreneurial ecosystem factors into SCCT-based digital entrepreneurship models, especially within emerging economy contexts.

### Practical implication

The findings of this study offer several practical implications for universities, educators, and policymakers seeking to foster digital entrepreneurial intention among students. First, the development of personal innovativeness should be approached as a gradual and supportive process rather than an imposed requirement. Educational environments that encourage experimentation, creativity, and problem-solving are more likely to cultivate innovativeness and, in turn, strengthen students’ confidence in their entrepreneurial capabilities. From a psychological standpoint, such environments may provide mastery experiences that reinforce efficacy beliefs and sustained motivational engagement.

Second, entrepreneurship education programs should integrate innovation-oriented activities with practical exposure to digital technologies. Training initiatives that emphasize hands-on learning, interdisciplinary collaboration, and the application of technology in entrepreneurial contexts can help students translate innovative thinking into perceived capability. Mechanisms that allow students to articulate, test, and refine entrepreneurial ideas, such as competitions, workshops, and industry-linked projects, may further support the development of digital entrepreneurial intention. These approaches may enhance students’ perceived control and competence, both of which are central to intention formation.

Third, given the pivotal role of digital entrepreneurial self-efficacy, educators should prioritize learning designs that enhance students’ sense of competence. Authentic learning experiences provide effective opportunities for strengthening both innovativeness and self-efficacy [40, 55]. Such experiences enable students to engage directly with entrepreneurial tasks, thereby reinforcing their confidence in managing digital ventures. Strengthening perceived capability may be particularly important in contexts characterized by rapid technological change and uncertainty.

Finally, the growing influence of artificial intelligence calls for its thoughtful integration into higher education curricula. Students across disciplines need to develop a balanced understanding of AI, including its capabilities, limitations, and ethical considerations. Instruction related to AI should remain objective and practice-oriented, enabling students to leverage AI tools without becoming overly dependent on them. At the institutional level, clear guidelines regarding the appropriate use of AI in learning and entrepreneurial activities may help prevent overreliance while preserving students’ intrinsic motivation for skill development. Careful integration of AI may support confidence-building without undermining autonomous competence development.

From a policy perspective, the findings suggest that governments and higher education authorities in emerging economies should strengthen digital transformation strategies that support entrepreneurial learning and AI accessibility among young people. Investments in digital infrastructure, AI-supported educational platforms, startup incubation programs, and university–industry collaboration may help students develop stronger entrepreneurial confidence and digital opportunity recognition capabilities.

The findings also highlight the importance of entrepreneurial ecosystem development. Universities, policymakers, digital platforms, and industry partners should work together to create supportive ecosystems that facilitate access to mentorship, entrepreneurial networks, digital resources, and innovation-oriented learning environments. Such ecosystem conditions may help students translate entrepreneurial intention into future entrepreneurial behavior and participation in the digital economy.

More broadly, fostering digital entrepreneurial intention among university students may contribute to long-term innovation capacity, digital workforce development, and sustainable economic growth in emerging economies. As digital transformation continues to reshape labor markets and entrepreneurial activities, strengthening students’ confidence and capability in digital entrepreneurship may generate broader societal and economic benefits through increased innovation, technology adoption, and startup participation.

### Limitation and future research direction

Despite its contributions, this study is subject to several limitations that suggest avenues for future research. First, the use of cross-sectional data limits the ability to capture the dynamic evolution of cognitive processes over time. Given the rapid pace of technological change, longitudinal research designs could provide deeper insights into how innovativeness, self-efficacy, and AI adoption interact throughout different stages of entrepreneurial development. Future studies may also explore reciprocal relationships between efficacy beliefs and technology use over time.

Second, while SCCT emphasizes the interaction between individual cognition and contextual factors, this study did not explicitly incorporate external influences such as educational environments or broader business conditions. Future studies could integrate both subjective cognitive factors and objective contextual variables to develop a more comprehensive understanding of digital entrepreneurial intention formation. Examining these interactions across different institutional and cultural settings may further enrich knowledge of entrepreneurship in emerging economies.

Third, this study did not consider several potential control variables, such as prior entrepreneurial experience, socio-economic background, and digital literacy. These factors may influence how individuals develop entrepreneurial cognition and intention within digital environments. Therefore, future research should incorporate a broader range of control variables to examine whether and how the observed relationships may vary under different individual and contextual conditions. Including these variables would provide a more comprehensive understanding of digital entrepreneurial intention and improve the generalizability and robustness of the findings. Regarding methodological considerations, while full collinearity VIF values indicated no serious common method bias concerns, future research may consider applying the marker variable technique for additional assurance.

Finally, this study was conducted in Vietnam and primarily focused on university students. As a result, the findings may not fully generalize to students in other countries, given that the role of AI adoption, digital self-efficacy, and entrepreneurial cognition may vary across societies with different levels of digital infrastructure, institutional support, and cultural context. Moreover, because the sample consisted mainly of students, the findings may not be generalizable to non-student populations, such as working professionals, entrepreneurs, or older individuals, who may possess different levels of practical experience, financial resources, and career motivations. Future studies are therefore encouraged to conduct cross-cultural comparisons and include more diverse demographic groups in order to validate and extend the applicability of the proposed framework across broader populations and contexts.

## Supplementary Information

Supplementary Material 1.

Supplementary Material 2.

## Data Availability

The datasets generated and analyzed during the current study are not publicly available due to confidentiality agreements with participants. However, the data supporting the findings of this study are available from the corresponding author upon reasonable request. Interested researchers can contact the corresponding author, Nguyen Thi Hoa Hong, via email at hongnth@ftu.edu.vn to request access.
