# Supplementary material for: Personal Innovativeness and Digital Entrepreneurial Intention: A Social Cognitive Perspective and the Moderating Role of AI Adoption
Source: BMC Psychol. 2026 Jul 22;14:1493. doi: 10.1186/s40359-026-05177-y (PMC13673797; doi:10.1186/s40359-026-05177-y)
Supplement: Supplementary file 1 [file 40359_2026_5177_MOESM1_ESM.docx]

| **Supplementary File 1 – English Questionnaire** | | | |
| --- | --- | --- | --- |
| **Construct** |  | **Items** | **Sources** |
| Digital Entrepreneurial Intention  (DEI) | DEI1 | My professional goal is to become an entrepreneur on digital platforms | Pham, M., Lam, B. Q., Nguyen, A. T. T., Dinh, T. K. T., & Tran, H. Y. (2024). How do environment and innovativeness affect digital entrepreneurial intention through education? An explanation from the social cognitive career theory. *Social Sciences & Humanities Open*, *10*, 101179. |
|  | DEI2 | I have very seriously thought of starting a digital business |  |
|  | DEI3 | I am determined to create a digital business in the future |  |
|  | DEI4 | I will make every effort to start and run my own digital business |  |
|  | DEI5 | I am ready to do anything to be an entrepreneur on digital platforms |  |
| Digital Entrepreneurial Self-efficacy  (SE) | SE1 | I am confident in digital-entrepreneurship | Pham, M., Lam, B. Q., Nguyen, A. T. T., Dinh, T. K. T., & Tran, H. Y. (2024). How do environment and innovativeness affect digital entrepreneurial intention through education? An explanation from the social cognitive career theory. *Social Sciences & Humanities Open*, *10*, 101179. |
|  | SE2 | I can control the creation process of digital-entrepreneurship |  |
|  | SE3 | I know the necessary practical details for digital-entrepreneurship |  |
|  | SE4 | I would have a high probability of succeeding in digital-entrepreneurship |  |
|  | SE5 | Digital-entrepreneurship would be easy for me |  |
|  | SE6 | I can become a digital-entrepreneur when I want |  |
| Personal Innovativeness (IN) | IN1 | I like to experiment with new information technology | Pham, M., Lam, B. Q., Nguyen, A. T. T., Dinh, T. K. T., & Tran, H. Y. (2024). How do environment and innovativeness affect digital entrepreneurial intention through education? An explanation from the social cognitive career theory. *Social Sciences & Humanities Open*, *10*, 101179. |
|  | IN2 | I would not hesitate to try out new information technology |  |
|  | IN3 | Among my fellows, I am usually the first to try out new information technology |  |
| AI adoption  (AI) | AI1 | I use AI tools to obtain information on the benefits realized in the area of digital entrepreneurship. | Duong, C. (2024). ChatGPT adoption and digital entrepreneurial intentions: An empirical research based on the theory of planned behaviour. *Entrepreneurial Business and Economics Review*, *12*(2), 129-142. |
|  | AI2 | I obtain information about entrepreneurial opportunities offered by government agencies/individuals through AI tools. |  |
|  | AI3 | I use AI tools to obtain information on loan deals for digital entrepreneurship. |  |
|  | AI4 | I can increase lots of knowledge about digital entrepreneurship through AI tools adoption. |  |
|  | AI5 | I use AI tools to obtain information about the problems and obstacles that will be encountered in the area of digital entrepreneurship. |  |
