# Supplementary material for: Personal Innovativeness and Digital Entrepreneurial Intention: A Social Cognitive Perspective and the Moderating Role of AI Adoption
Source: BMC Psychol. 2026 Jul 22;14:1493. doi: 10.1186/s40359-026-05177-y (PMC13673797; doi:10.1186/s40359-026-05177-y)
Supplement: Supplementary file 2 [file 40359_2026_5177_MOESM2_ESM.docx]

**Supplementary File 2 – CB-SEM Measurement Model Comparison**

This supplementary file presents the measurement model results estimated using the CB-SEM algorithm, provided for comparison with the PLS-SEM results reported in the main manuscript. As noted in Section 3.4, CB-SEM is not appropriate for estimating the full structural model in this study due to the presence of product indicator-based interaction terms. The comparison is therefore limited to the measurement model only.

As shown in Table S2, reliability indices are consistent across both methods, with all CA, rho_A, and CR values exceeding the recommended threshold of 0.70. Factor loadings are statistically significant for all constructs under both approaches. Two constructs, AI adoption (0.460) and Self-efficacy (0.478), show AVE values marginally below the conventional threshold of 0.50 under CB-SEM estimation. However, given that all loadings remain significant and CR values are satisfactory, this is considered acceptable and is consistent with the known tendency of CB-SEM to produce more conservative variance estimates compared to PLS-SEM under conditions of moderate indicator communality (Hair et al., 2019).

**Table S2: Measurement model comparison - PLS-SEM vs. CB-SEM**

| **Construct** | **PLS-SEM CA** | **PLS-SEM rho_A** | **PLS-SEM CR** | **PLS-SEM AVE** | **PLS-SEM Loadings** | **CB-SEM CA** | **CB-SEM CR** | **CB-SEM AVE** | **CB-SEM Loadings** |
| --- | --- | --- | --- | --- | --- | --- | --- | --- | --- |
| AI adoption (AI) | 0.810 | 0.813 | 0.868 | 0.568 | 0.737 - 0.769 | 0.810 | 0.810 | 0.460 | 0.656 - 0.704 |
| Digital entrepreneurial intention (DEI) | 0.825 | 0.834 | 0.876 | 0.587 | 0.743 - 0.789 | 0.825 | 0.966 | 0.851 | 0.918 - 0.934 |
| Personal innovativeness (IN) | 0.769 | 0.770 | 0.867 | 0.685 | 0.805 - 0.851 | 0.769 | 0.770 | 0.532 | 0.646 - 0.822 |
| Digital entrepreneurial self-efficacy (SE) | 0.846 | 0.847 | 0.886 | 0.565 | 0.740 - 0.764 | 0.846 | 0.846 | 0.478 | 0.655 - 0.734 |

**Reference**

Hair, J. F., Risher, J. J., Sarstedt, M., & Ringle, C. M. (2019). When to use and how to report the results of PLS-SEM. *European Business Review*, 31(1), 2–24. <https://doi.org/10.1108/EBR-11-2018-0203>.
